# Complex Roles of NEIL1 and OGG1: Insights Gained from Murine Knockouts and Human Polymorphic Variants

**DOI:** 10.3390/dna2040020

**Published:** 2022-12-01

**Authors:** R. Stephen Lloyd

**Affiliations:** Oregon Institute of Occupational Health Sciences, Department of Molecular and Medical Genetics, Oregon Health & Science University, Portland, OR 97239, USA;

**Keywords:** base excision repair, oxidatively induced DNA damage, AP lyase, glycosylase catalytic mechanisms, DNA repair knockout models, metabolic syndrome, single-nucleotide variants, aflatoxin, acute myeloid leukemia, hepatocellular carcinomas

## Abstract

DNA glycosylases promote genomic stability by initiating base excision repair (BER) in both the nuclear and mitochondrial genomes. Several of these enzymes have overlapping substrate recognition, through which a degree of redundancy in lesion recognition is achieved. For example, OGG1 and NEIL1 both recognize and release the imidazole-ring-fragmented guanine, FapyGua as part of a common overall pathway to cleanse the genome of damaged bases. However, these glycosylases have many differences, including their differential breadth of substrate specificity, the contrasting chemistries through which base release occurs, the subsequent steps required to complete the BER pathway, and the identity of specific protein-binding partners. Beyond these differences, the complexities and differences of their in vivo biological roles have been primarily elucidated in studies of murine models harboring a knockout of *Neil1* or *Ogg1*, with the diversity of phenotypic manifestations exceeding what might have been anticipated for a DNA glycosylase deficiency. Pathologies associated with deficiencies in nuclear DNA repair include differential cancer susceptibilities, where *Ogg1*-deficient mice are generally refractory to carcinogenesis, while deficiencies in *Neil1*-deficient mice confer cancer susceptibility. In contrast to NEIL1, OGG1 functions as a key transcription factor in regulating inflammation and other complex gene cascades. With regard to phenotypes attributed to mitochondrial repair, knockout of either of these genes results in age- and diet-induced metabolic syndrome. The adverse health consequences associated with metabolic syndrome can be largely overcome by expression of a mitochondrial-targeted human OGG1 in both wild-type and *Ogg1*-deficient mice. The goal of this review is to compare the roles that NEIL1 and OGG1 play in maintaining genomic integrity, with emphasis on insights gained from not only the diverse phenotypes that are manifested in knockout and transgenic mice, but also human disease susceptibility associated with polymorphic variants.

## 1. Introduction

The identification of enzymes associated with the initiation of BER of oxidatively induced DNA damage was first established in *Escherichia coli*, comprising formamidopyrimidine DNA glycosylase (Fpg), endonuclease III (Nth1), endonuclease VIII (Nei), and adenine:guanine DNA glycosylase (MutY) (reviewed in [1,2]). In humans, three genes have been described for encoding NEI-like glycosylases (NEIL) (reviewed in [3]), and their initial characterizations were concurrently described in multiple laboratories [4–7]. The first eukaryotic functional equivalent of *E. coli* Fpg was discovered by functional complementation of a strain deficient in both Fpg and MutY. This strategy identified the *Saccharomyces cerevisiae OGG1* gene (*y*OGG1), which facilitated the initial biochemical characterization of this enzyme [8]. Building on this discovery and characterization, the human BER enzyme that initiates repair of 8-oxoG (hOGG1) was concurrently reported in multiple laboratories [9–15]. This review focuses on in vitro investigations of human NEIL1 and OGG1, as well as in vivo phenotypic consequences following knockout of murine *Neil1* and *Ogg1*, including organelle-specific complementation. When possible, extrapolations are made toward disease implications associated with deficiencies in human OGG1 and NEIL1.

## 2. Target Site Location and Substrate Recognition

**Figure 1.**
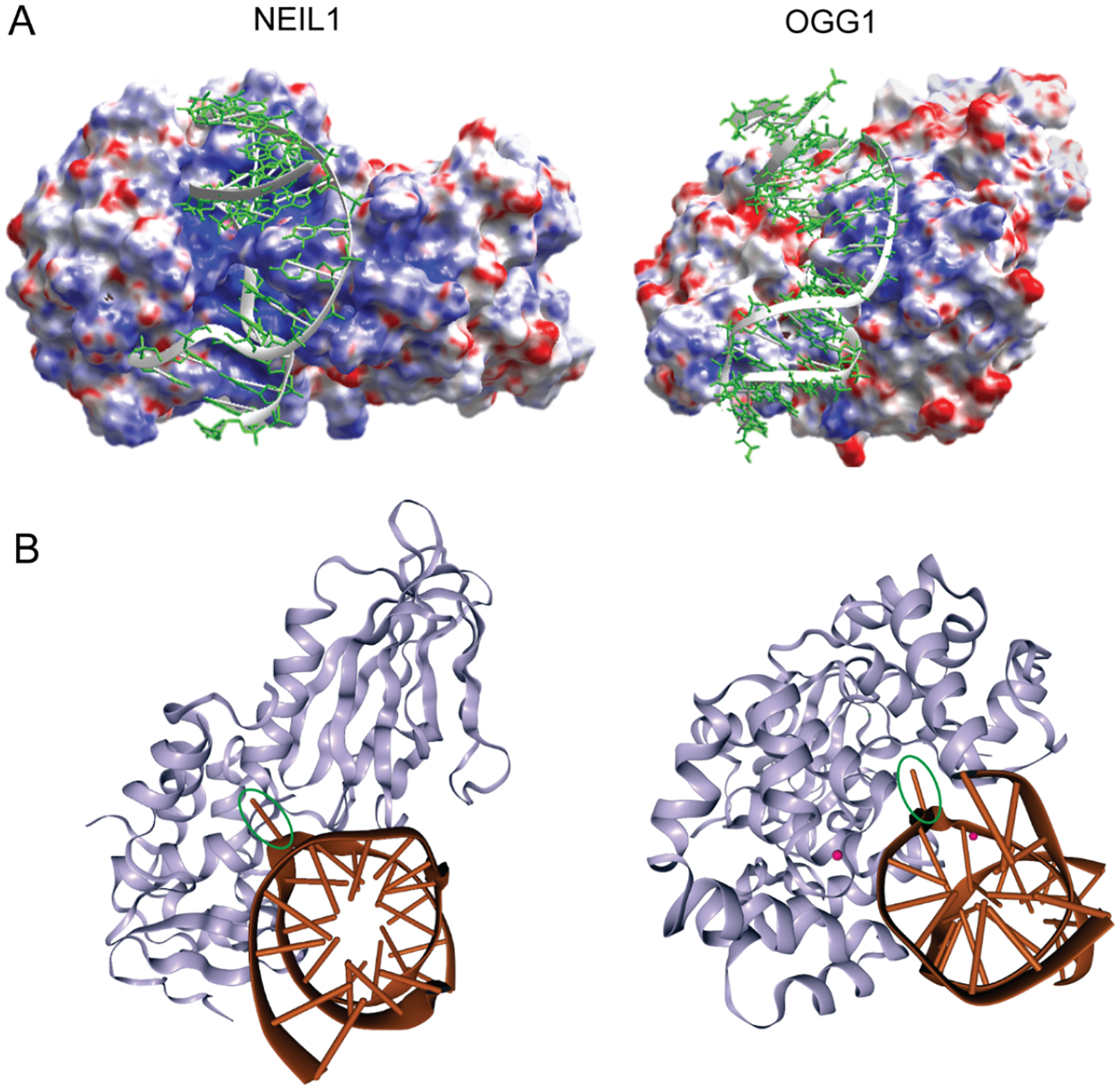
Charge distribution on the surface (**A**) and nucleotide flipping (**B**) in co-crystal structures of DNA glycosylases: The crystallographic coordinates of NEIL1 and OGG1 bound to lesion-containing DNA were retrieved from the Protein Data Bank (rcsb.org, last accessed date: 27th February 2020; PDB IDs 5ITX [20] and 1YQR [17], respectively). The images of Poisson–Boltzmann electrostatic potential surface maps were generated using the Schrödinger package (schrodinger.com). The surface is colored according to electrostatic potential (positive: blue; negative: red). The images of glycosylase–DNA complexes were created with NGL Viewer [33] at the RCSB PDB site (rcsb.org). The damaged nucleotides (encircled) are at extrahelical positions. This figure was adapted from [34].

As is common among DNA glycosylases, X-ray-derived crystal structures of both the apoenzymes and the DNA co-crystal complexes of NEIL1 and OGG1 reveal that these relatively low-molecular-weight proteins share an asymmetric surface charge distribution through which nonspecific DNA binding and processivity are achieved (Figure 1A) [16–23]. This differential surface charge distribution is believed to guide short-range enzyme–DNA orientation geometries, leading to productive—albeit transient—nonspecific DNA binding. Once properly oriented on DNA, non-directional sliding along DNA provides the context through which alterations in base structure and helical stability can be detected through partial nucleotide flipping. This mechanism of nonspecific DNA sliding and the biological importance of this nonspecific binding was first described for the DNA glycosylase known as T4 pyrimidine dimer glycosylase (T4-pdg) [24–27], and it has subsequently been described for many DNA glycosylases (reviewed in [28–32]). Moreover, as revealed by various biophysical techniques and structure–function analyses, these enzymes create a flexible active site into which damaged nucleotides can be flipped and accommodated between globular and active site-forming domains (Figure 1B) [17,19,20,22,23]. Specific binding is achieved through this complex set of molecular motions, bringing active site residues in close proximity to the damage-containing nucleotides to initiate the chemistry of glycosyl bond scission. The specific chemical mechanisms for base release generally dictate whether a glycosylase will only catalyze base release, leaving an abasic (AP) site with the phosphodiester backbone intact, or whether both base release and phosphodiester incision are mechanistically coupled. These are discussed in the following section, in which the chemical mechanisms of OGG1 and NEIL1 are contrasted.

Investigations reporting the initial cloning, expression, and characterization of NEIL1, NEIL2, and NEIL3 generally utilize synthetic oligodeoxynucleotides containing site-specific base damage—including dihydrouracil, thymine glycol, and other saturated pyrimidines—to identify substrate specificities of NEIL1 [4–7]. Based on data demonstrating that NEIL1 expression was correlated well with the S-phase of the cell cycle [5,35,36], it was hypothesized that it may possess activity at sites of stalled replication, as modelled through DNA bubble structures. In agreement with this hypothesis, in vitro biochemical studies demonstrated that NEIL1 did not require fully double-stranded DNAs as substrates, but that it can catalyze incision on substrates located within designed bubble or forked structures as well as single-stranded DNA [37–43]. In some cases, rates of incision were accelerated using these substrate designs. NEIL1’s association with proteins associated with replication complexes is described below in Section 4. Although these investigations using synthetic oligodeoxynucleotides provide a framework from which to evaluate the base specificity of NEIL1, investigations by Dr. Miral Dizdaroglu and collaborators have generated the most comprehensive insights into the specificity of NEIL1 by using DNA damaged with γ-rays followed by GC/MS analyses [5,44–46]. This experimental design measures base release following incubation of purified NEIL1 with irradiated, high-molecular-weight genomic DNA. These data reveal that, in addition to various saturated pyrimidines—including thymine glycol, 5-OH-cytosine, 5,6-dihydrouracil, and 5-OH uracil—the most preferred substrates for NEIL1 are the two ring-fragmented purines 2–6-diamino-4-hydroxy-5-formamidopyrimidine (FapyG) and 4,6-diamino-5-formamidopyrimidine (FapyAde). In subsequent years, NEIL1 has been shown to recognize and incise DNA at all tested alkyl-modified Fapy-dG substrates, including methyl Fapy-dG, nitrogen mustard Fapy-dG, and aflatoxin B_1_ (AFB_1_)-Fapy-dG [43,46–49]. In addition to ring-fragmented and alkyl-FapyGua purines, a number of studies have demonstrated that the DNA substrates with the highest catalytic efficiencies for NEIL1 are the secondary oxidation products of 8-oxodG—spiroiminohydantoin (Sp) and 5-guanidinohydantoin (Gh)—as well as a variety of Sp-amine adducts, including Sp-lysine, Sp-glucosamine (GlcN), and tetra- and pentapeptides [40,41,47,50–53]. NEIL1 also catalyzes the incision of DNA containing 5-carboxylcytosine as part of a substrate redundancy for thymine DNA glycosylase (TDG), in a repair process associated with active DNA demethylation [54]. Furthermore, NEIL1 has also been demonstrated to initiate repair of a variety of psoralen-induced interstrand DNA crosslinks [55–57].

In contrast to the extremely broad substrate range of NEIL1, to date, the only ionizing-radiation-induced base damages recognized by OGG1 are 7,8-dihydro-8-oxo-2′-guanine (8-oxoGua) and FapyGua [5,58,59]. The initial characterizations of human OGG1 demonstrated that there was a clear preference for the nucleotide opposite 8-oxo-dG, with near-exclusive incision when a dC was opposite the site of the lesion [9–15]. Given this dominant base-pair recognition preference, it is anticipated that OGG1 will not incise damage within single-stranded DNAs. Furthermore, rates of incision of synthetic oligodeoxynucleotides vary greatly depending on sequence context, methylation status, and the presence of mismatched nucleotides [60]. The substrate specificity of OGG1 can be modulated by carrying out reactions in the presence of the AP endonuclease APEX1, where OGG1 can release 8-oxoAde, 8-oxoIno, and 6-methoxy-8-oxoGua [61]. Since NEIL1 does not catalyze the release of 8-oxoGua, these enzymes only share a single substrate in common: FapyGua. Measurements of the catalytic efficiencies of the removal of FapyGua from duplex DNA are comparable: 9.3 ± 0.6 × 10^−5^ and 9.0 ± 0.2 × 10^−5^ min^−1^ mmol^−1^ L for NEIL1 and OGG1, respectively [45,62,63].

Overall, although NEIL1 and OGG1 only share a single common substrate, their combined spectrum of recognition includes the most abundant lesions and covers a major portion of the total oxidatively induced base damage, as can be inferred from the data using ionizing radiation as a source of oxidation. However, even though this review focuses on these two glycosylases, human cells have multiple DNA glycosylases that provide built-in redundancy in the recognition and repair of DNA base damage, including NEIL2, NEIL3, and NTH1. A rigorous analysis of the overlapping substrate specificities of these glycosylases, as well as several non-human glycosylases, has previously been reviewed [45].

## 3. Mechanisms of Chemical Catalysis with Implications for the Completion of the BER Pathway

**Figure 2.**
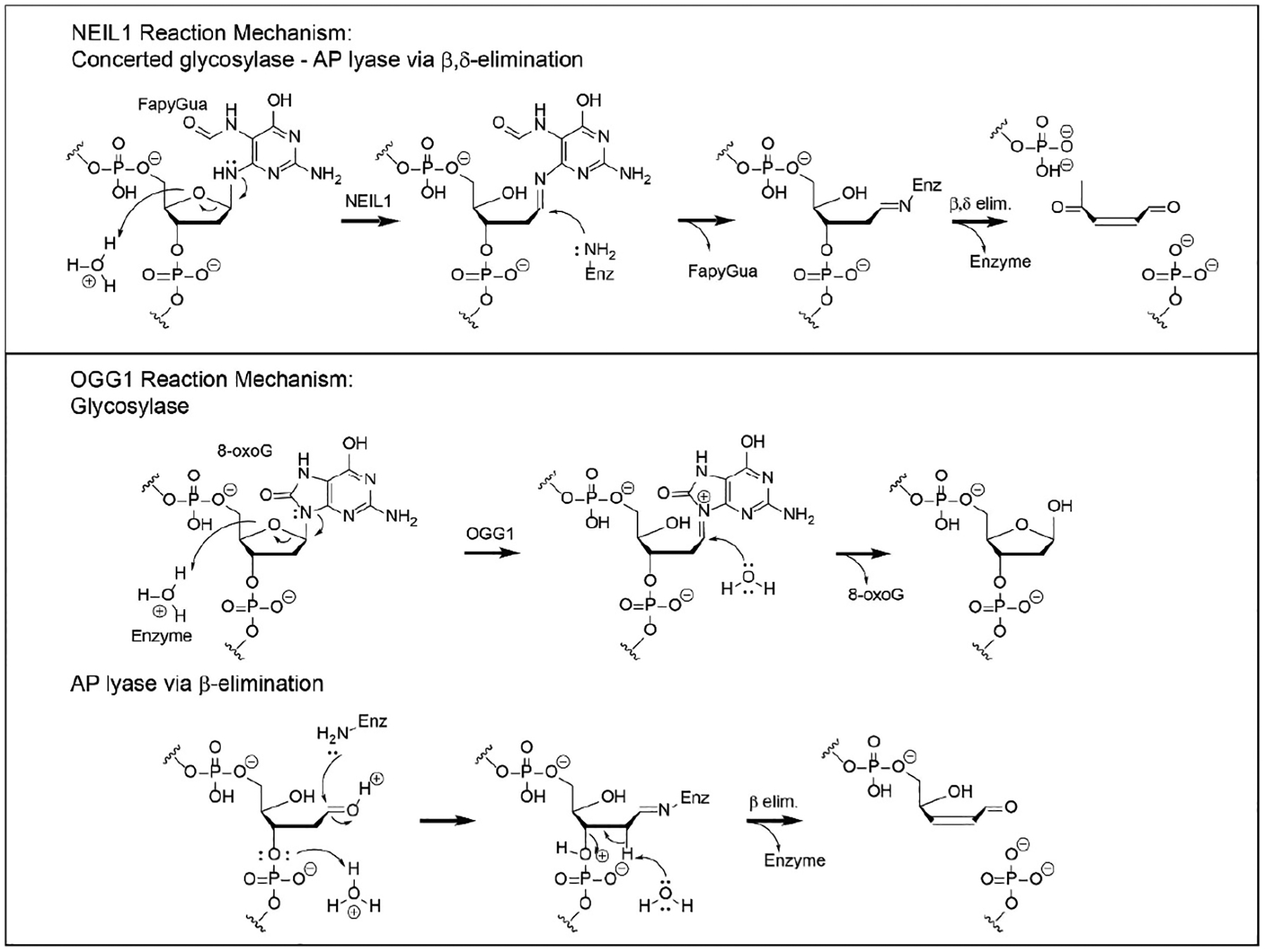
Chemistry of the mechanisms of NEIL1 and OGG1.

Although both NEIL1 and OGG1 are DNA glycosylases, the chemical mechanisms by which they catalyze base release are distinct (Figure 2). DNA glycosylases that also have an associated AP site lyase activity utilize either a primary or secondary amine to form a covalent imine intermediate with the C1′ of the deoxyribose that is associated with the damaged base. This mechanism was first established for T4-pdg, in which the enzyme–DNA intermediate could be trapped by a concomitant reaction with sodium borohydride [64–67]. Such covalent intermediates are stable under further biophysical analyses, facilitating the mapping of active site nucleophiles for all glycosylases with AP lyase activities [68,69]. Enzymes that catalyze a β-elimination reaction all use a primary amine (e.g., a lysine or an N-terminal α-amino group), while enzymes that catalyze β,δ-elimination reactions—such as NEIL1 and NEIL2—use the secondary amine of an N-terminal proline (P2). For NEIL1, the adjacent glutamic acid (E3) is essential for this reaction as a proton donor [4,23]. Site-directed mutagenesis studies of NEIL1 have also implicated Lys54 and Arg339 in the β-and δ-elimination reactions [37,70]. Additional insights into critical portions of the enzyme for catalysis have been generated by site-directed mutagenesis and in vitro characterization. Mutagenesis at R277 to R277A resulted in decreased glycosylase activity, while full lyase function was retained [16]. Characterization of two of the human polymorphic variants of NEIL1—Gly83Asp and Cys136Arg—showed no glycosylase activity [44,71]. The loss of function of Gly83Asp was attributed to the introduction of an aspartic acid residue at the entrance of the nucleotide-flipping pocket. The Cys136Arg variant is located in the flexible hinge region of NEIL1, where changes can affect the dynamic opening and closing of the substrate-recognition pocket.

OGG1 was originally characterized as a bifunctional glycosylase-AP lyase, based on sodium borohydride trapping of an enzyme–DNA intermediate. However, structure–function analyses have demonstrated that the glycosylase reaction is catalyzed by an activated water molecule, coordinated by Asp268 [72]. Thus, the glycosylase reaction is mechanistically independent of the β-elimination reaction, which occurs via the subsequent reaction of an opportunistically positioned lysine residue (K249) in close proximity to the AP site. This separation of function was confirmed by the creation of the site-directed mutants K249C/C253K, which retain full glycosylase activity while exhibiting no lyase function, and C253W, which is devoid of glycosylase activity but retains full AP lyase function. Thus, to be consistent with its actual catalytic mechanism, OGG1 should be referred to as a monofunctional glycosylase, with the capacity to also catalyze nicking at AP sites. Intracellularly, data have been reported that support the conclusion that OGG1 functions as a glycosylase without concomitant lyase activity. This was explicitly tested by engineering shuttle vectors containing site-specific 8-oxo-dG lesions that were flanked by either 5′ or 3′ nuclease-resistant phosphorothioate linkages [73]. This study revealed that, in cells, OGG1 did not catalyze strand incision, and that cleavage of the phosphodiester backbone 5′ to the lesion was carried out by APE1. This is consistent with prior in vitro biochemical studies that demonstrated that physiologically relevant concentrations of magnesium preferentially abrogated the AP lyase activity of OGG1 [74]. Additional support for OGG1 functioning as a monofunctional glycosylase is based on analyses of data from the infection of OGG1-proficient gastric epithelial cells with *Helicobacter pylori*, in which there were increased levels of AP sites, while suppression of OGG1 in the same experimental design resulted in decreased levels of AP sites [75].

Additional structure–function investigations have been reported concerning the potential roles of all cysteine residues in OGG1 in the context of its varied functions within cells [76]. These studies revealed that the majority of mutants affected either the glycosylase activity (C146 and C255), the lyase activity (C140, C163, C241, and C253), or the structural stability (C253), with mutagenesis at only two cysteine residues resulting in no discernable loss of function (C28 and C75). These data suggest that differential site-specific oxidation of cysteine residues in OGG1 could play a major regulatory role in switching between repair and transcriptional regulation.

Although cancer and cancer-associated variants are discussed below, a recent report by Popov et al. [77] utilized molecular dynamics analyses of 20 clinically observed variants of OGG1, combined with successful expression and structure–function analyses of 13 of these variants. Two of the variants—I145M and G202C—were severely catalytically compromised by >10-fold, while an additional three—R161W, V267M, and S292N—were reduced by 1.5–2-fold. It is also of interest that two of the variants exceeded WT rates, with H111R and R206C showing a 2.0–2.5-fold increase. Overall, their experimental data were in good agreement with the molecular dynamics simulations, emphasizing the utility of this strategy in large structure–function analyses and the potential identification of pathogenic variants of DNA glycosylases.

The individual downstream steps in BER are significantly different for repair initiated by NEIL1 and OGG1. As elaborated below, these differences are dictated by the nature of the chemistry through which base release and phosphodiester bond scission occur. Downstream repair of NEIL1-initiated repair builds on the single-nucleotide gapped DNA, with both 5′ and 3′ phosphates. Unlike monofunctional glycosylases, or glycosylase-AP lyases, there is no requirement for the activity of an AP endonuclease (APE1); rather, the 3′-phosphate removal is catalyzed by the activity of DNA kinase/phosphatase (PNKP), creating a 3′-OH [78]. In fact, APE1 is unable to remove a 3′phosphate. Thus, PNKP activity generates the ideal substrate for the filling of single-nucleotide gaps by DNA polymerase β and subsequent ligation by DNA ligase 3 [79].

In contrast, OGG1-initiated repair will result in either an AP site within fully duplex DNA or base loss and β-elimination generating a 3′-dRp. Both of these intermediates require processing by APE1 to create the 3′-OH and subsequent reaction(s) by DNA polymerase β to remove the 5′ sugar moiety via its 8 kDa AP lyase domain if OGG1 functions as a glycosylase alone. The resulting gap is filled and sealed as described above.

## 4. Cell-Cycle Specificity and Protein-Binding Partners

Early characterization of NEIL1-mediated repair revealed a predominant S-phase cell-cycle specificity, suggesting an involvement and association with genomic replication [5,35,36]. This hypothesis was supported by biochemical studies that showed the ability of NEIL1 to excise base damage from bubble and forked structures [37–43]. A large number of proteins have been identified as associated with NEIL1, including replication- and repair-associated proteins, e.g., PCNA, RPA, RFC, heterogeneous nuclear ribonucleoprotein U, Werner syndrome protein, PARP1, FEN1, DNA ligase 3, polymerase δ, polymerase β, NEIL2, TDG, HDAC1, and the Rad9–Rad1–Hus1 complex [38,39,42,54,78,80–83].

Unlike *NEIL1*, on which there is an abundance of experimental evidence supporting cell-cycle regulation, the literature concerning cell-cycle regulation of *OGG1* is conflicting. As part of the original cloning and characterization of the essential promoter of *OGG1*, it was shown that there were no expression differences across any phases of the cell cycle and that there were equivalent levels of 8-oxodG nicking activity across all phases [84]. Additionally, Western blot analyses revealed no changes in OGG1 levels throughout the cell cycle [85]. However, these conclusions are at odds with another study showing a cell-cycle dependence of expression of the two major isoforms of OGG1 and a dynamic re-localization to the nucleolus during the S-phase [86]. It is not clear what accounts for this underlying discrepancy.

As was the case for substrate specificities, the protein-binding partners of OGG1 share almost no overlap with the NEIL1-associated proteins but include the repair-associated proteins PARP1, XRCC1, NEIL1, APE1, RECQL4, UV-DDB, CUX1, CUX2, SATB1, and the Rad9–Rad1–Hus1 complex [61,87–99]. The protein-binding partners of OGG1 and the significance of these interactions have been comprehensively reviewed [100]. Recently, a role for OGG1 has been discovered in the regulation of Myc-controlled genes, in which direct binding of Myc to OGG1 resulted in an inhibition of OGG1 incision activity and recruitment of Myc to its promoters [101]. These data are consistent with the hypothesis that catalytically inactive forms of OGG1 bind to 8-oxo-dG sites in promoter regions, thereby influencing the activation of inducible transcriptional networks. This is discussed in more detail in Section 5 below.

In addition to their nuclear-localized binding partners, both NEIL1 and OGG1 interact with mitochondrial-imported proteins that are associated with repair and replication. mtNEIL1 binds to the tetrameric form of mitochondrial single-stranded binding protein (mtSSB) in the presence of duplex DNA, but in the absence of DNA the complex is reduced to a NEIL1–mtSSB complex [102]. Furthermore, mtNEIL1 also interacts with mitochondrial transcription factor A (TFAM) [103]. A proposed biological role for this interaction is that in the absence of NEIL1, TFAM-transcribed mitochondrial genes are significantly downregulated.

ERCC6 (CSA) and ERCC8 (CSB) also bind mitochondrial OGG1 [104]. The interactions of mtOGG1 with CSA and CSB protect against age- and stress-induced mtDNA mutations, as well as apoptosis-mediated loss of subcutaneous fat.

## 5. Phenotypes in Knockout Mice

Given the central roles that NEIL1 and OGG1 play in both nuclear and mitochondrial genome maintenance, it was anticipated that the loss of these critical functions would result in an increased frequency of cellular transformation and carcinogenesis. Although not originally anticipated, changes and/or reductions in mitochondrial genome integrity resulted in metabolic disorders and disruptions in inflammatory responses in oxidatively stressed organs such as the lungs, brain, liver, stomach, and colon. Several decades of investigations have laid the basic foundation for developing a clearer understanding of the adverse consequences that result from the loss of these enzymes.

### 5.1. Cancers

Among the most perplexing observations using murine knockout models of *NEIL1* and *OGG1* deficiencies are the differences in cancer susceptibilities. *Ogg1*^−/−^ mice are refractory to spontaneous carcinogenesis. These data are extraordinary in that the loss of BER-initiated repair of 8-oxo-dG lesions and subsequent replication bypass of residual 8-oxo-dG lead to high-frequency misincorporation of dA opposite the oxidized G. However, if the misincorporated dA is not removed by the adenine DNA glycosylase MutY-homologue (MUTYH), the subsequent round of replication generates a stable, inheritable G-to-T transversion. Thus, it was not surprising that knockout of both *Ogg1*^−/−^ and *Myh*^−/−^ in murine models led to increased lung and small intestinal cancers [105,106]. In addition to the lack of spontaneous carcinogenesis, even under the most extreme oxidizing conditions associated with potassium bromate ingestion through supplementation of drinking water and 8-oxo-dG levels increasing >1000-fold over baseline, no carcinogenesis was observed in *Ogg1*^−*/*−^ mice [107]. Furthermore, in our own laboratory’s experience with two independently derived *Ogg1*^−*/*−^ mouse strains, no tumors were ever noted, whether in mice raised under conditions that induce many other physiological alterations—such as a high-fat diet—or in mice that achieved ages > 20 months. This absence of a carcinogenic phenotype with concomitant mutagenesis has not been adequately rationalized but may be related to the absence of an induction of NF-κB-associated inflammation.

In contrast to the results described above, and given the substrate specificity of OGG1, it is of interest that *Ogg1*^−*/*−^ mice showed enhanced multiorgan carcinogenesis when challenged with combinations of a variety of DNA-alkylating agents, including nitrosamines and nitrosourea [108]. Although it was speculated that these alkylating agents enhanced oxidative stress, thereby accounting for increased carcinogenesis in multiple sites, this was not explicitly tested. Furthermore, *Ogg1*^−*/*−^ mice have also been shown to have increased susceptibility to phenobarbital- and UVB-induced carcinogenesis [109,110].

In contrast to the lack of spontaneous carcinogenesis observed in *Ogg*^−*/*−^ mice, *Neil1*^−*/*−^ mice show age- and gender-dependent spontaneous carcinogenesis—primarily in the liver and lungs [111,112]. In mice aged over a year, spontaneous formation of lung adenomas and adenocarcinomas was observed in 12% of the males and 0% of females, while hepatocellular carcinomas were found in 16% of males and 11% of females [112]. Creation of a double knockout of the *Neil1* and *Nth1* genes resulted in significant increases in the spontaneous frequency and distribution of tumors in both male and female mice relative to either individual knockout [112]. In males, lung adenomas and adenocarcinomas were observed in 74% and 41% of males and females, respectively, while hepatocellular carcinomas increased to 47% and 17% in males and females, respectively. These studies suggest an underlying genomic instability in *Neil1*^−*/*−^ mice that can result in carcinogenic endpoints. However, the studies described above have not been adequately reconciled with independently constructed knockouts of *Neil1*, double knockouts of *Neil1* and *Neil2*, or the triple knockout of all of the murine *Neil* genes, in which all three sets of knockouts showed no increases in cancers, mutations, or oxidatively induced base damage [113]. Although there are no studies resolving these differences in spontaneous carcinogenesis, it is possible that additional factors—such as differences in the intestinal microbiome—could influence these rates.

As previously described, the substrate specificity of NEIL1 encompasses a variety of alkylated FapyGua adducts, including AFB_1_-FapyGua [43]. To test the in vivo significance of Neil1-initiated BER of aflatoxin-induced DNA damage, *Neil1*^−*/*−^ mice and control C57Bl6 mice were challenged with two concentrations of AFB_1_, and their adduct accumulation was measured at 6 and 48 h post-exposure, while induction of hepatocellular carcinogenesis was measured at 15 months [43]. These analyses revealed a threefold increase in the accumulation of AFB_1_-FapyGua adducts in *Neil1*^−*/*−^ mice versus control C57Bl6 mice at 48 h post-injection of aflatoxin. This differential accumulation was attributed to the loss of BER-initiated repair, even though NER repair was active. As hypothesized for the carcinogenesis portion of this study, and in agreement with the elevated levels of AFB_1_-FapyGua accumulation in *Neil1*^−*/*−^ mice, the frequency of hepatocellular carcinoma (HCC) was greatly increased in these mice relative to their wild-type counterparts. Using tumor burden as a relative risk assessment, *Neil1*^−*/*−^ mice displayed a 3.4-fold increase in HCC formation relative to control mice receiving comparable doses of aflatoxin. Previously, in a separate investigation, the cancer risk associated with the loss of nucleotide excision repair following aflatoxin exposure was measured in *XPA*^−*/*−^ mice [114]. Comparable analyses of HCC formation in aflatoxin-treated *XPA*^−*/*−^ versus control wild-type mice revealed a modest 1.2-fold increase in liver carcinogenesis. Thus, at least in murine models, BER-initiated repair appears to be significantly more important in suppressing aflatoxin-induced HCCs than NER.

Extending studies on cancer susceptibility in *Neil1*^−*/*−^ mice, and given NEIL1’s role in the recognition and repair of oxidatively induced base damage, it was hypothesized that deficiencies in Neil1 could confer increased sensitivity to UVB-induced carcinogenesis via increased levels of reactive oxygen species [115]. In response to long-term but low-level UVB exposure, *Neil1*^−*/*−^ mice showed much greater levels of cutaneous tissue destruction and enhanced chronic inflammatory responses as measured by cytokine message levels, along with higher levels of neutrophil infiltration, relative to age-matched controls. However, in this study, differential ca rcinogenesis assessments could not be performed, due to the severity of tissue loss in the *Neil1*^−*/*−^ mice.

### 5.2. Metabolic Syndrome

The term metabolic syndrome, which is also called insulin resistance syndrome, refers to a group of conditions that, on aggregate, raise an individual’s risk of coronary heart disease, type 2 diabetes, stroke, and other diseases. It is characterized by abdominal obesity, high blood pressure, high blood sugars and triglycerides, and low HLD cholesterol.

Although our laboratory’s original justification for creating a whole-body knockout of *Neil1* was in anticipation of elevated mutagenesis and carcinogenesis due to decreased repair of oxidatively induced DNA damage, the most dominant phenotype in the *Neil1*^−*/*−^ mice was the development of midlife metabolic syndrome [111]. Even on a standard low-fat chow diet, male mice older than 9 months of age were preferentially susceptible to the accumulation of massive amounts of fat within the subcutis, retroperitoneum, mediastinum, and abdomen. Livers were enlarged, granulated, and pale yellow, with >50% of the cellular contents being a mixture of micro- and macrovesicular fat globules. The most prominent areas in the liver for fat accumulation were the centrilobular regions. Furthermore, livers showed no overt indication of significant inflammation or fibrosis. Although the increase in weight gain was readily discernable beyond 9 months, analyses of circulating leptin levels were clearly evident at 3 months of age, proving to be a strong predictor of future weight gains and fatty liver disease. Since *Neil1* is differentially spliced for both nuclear and mitochondrial localization, we hypothesized that a potential driver of metabolic syndrome in these mice could be the accumulation of mitochondrial DNA (mtDNA) damage and deletions. Steady-state levels of mtDNA damage were assessed by long-range PCR, in which PCR-blocking lesions were calculated by reductions in full-length (16 kb) mtDNA, and deletions (less than full-length mtDNAs) were visualized by reducing the time of the PCR extension reactions to selectively amplify these molecules. For both criteria, *Neil1*^−*/*−^ liver samples showed consistent evidence of increased damage and progressive loss of mtDNA integrity [111]. Such a loss in mitochondrial genomic integrity would be expected to compromise oxidative phosphorylation efficiencies which, in turn, would further amplify the production of reactive oxygen species and increase the DNA damage burden. Although not explicitly tested in these studies, these results suggest that correction of the mitochondrial repair deficiency by way of transgenic expression of a mitochondrial-targeted NEIL1 could prevent metabolic syndrome in *Neil1*^−*/*−^ mice.

The previous investigation exclusively focused on phenotypes in aged *Neil1*^−*/*−^ mice that consumed a low-fat diet. In a follow-up study, young *Neil1*^−*/*−^ and wild-type mice were challenged with a high-fat diet, and weight gain and other readouts of metabolic syndrome were analyzed [116]. Consistent with the conclusions from the original report, *Neil1*^−*/*−^ mice gained significantly more weight and had more severe hepatic steatosis relative to control mice and showed decreased mtDNA and protein content. The high-fat-diet-fed *Neil1*^−*/*−^ mice also showed elevated inflammatory gene expression profiles relative to their wild-type counterparts, which may also be an indicator of this genotype having a lowered threshold for tolerance of cellular oxidative stress.

Since *Neil1*^−*/*−^ mice showed hallmarks of susceptibility to the development of metabolic syndrome, we also investigated whether this phenotype was restricted to *Neil1*^−*/*−^ mice or whether *Ogg1*^−*/*−^ mice would also manifest a similar phenotype. Consistent with the phenotypes observed in *Neil1*^−*/*−^ mice, on a low-fat diet, aged *Ogg1*^−*/*−^ mice were significantly fatter and heavier than their control counterparts [117]. Also consistent with prior studies, a 10-week high-fat diet challenge of young, age-matched mice revealed that the *Ogg1*^−*/*−^ mice also experienced greater weight gain, increased adiposity, and fatty livers, even though their food intake and physical activity were not significantly different. This weight gain was accompanied by concomitant hyperinsulinemia and impaired glucose tolerance, suggesting the development of insulin resistance and, potentially, a prediabetic condition. Indirect calorimetric measurements revealed that the *Ogg1*^−*/*−^ mice showed characteristic changes from fat oxidation towards carbohydrate utilization—a result that was consistent with their obese phenotype. These data were supported by gene expression profiles indicating decreased hepatic fat oxidation. While these studies focused on hepatic alterations associated with metabolic syndrome, follow-up investigations examined whether skeletal muscle was comparably affected [118]. The *Ogg1*^−*/*−^ mice showed significant increases in the accumulation of triacylglycerol, cholesterol esters, and diacylglycerols in the skeletal muscle. Unlike the analyses of the liver, the skeletal muscle did not show decreased levels of mtDNA or increased 8-oxo-dG. This investigation also revealed that *Ogg1*^−*/*−^ mice showed a large decrease in grip strength and reduced treadmill endurance.

In addition to tissue-specific control and manifestations of fat accumulation, there is an abundance of literature that associates changes in the composition of the intestinal microbiome with the development of obesity phenotypes [119]. In contrast to wild-type mice, *Ogg1*^−*/*−^ mice fed either a normal chow or a high-fat diet showed a significantly greater microbial diversity on either diet [120]. These analyses revealed that *Ogg1*^−*/*−^ mice retained a microbiota that was consistent with increased energy harvest—a result that is highly consistent with the previously described obesity phenotype. In addition, the microbiome of the *Ogg1*^−*/*−^ mice also showed significant increases in pro-inflammatory microbes, even though the airway pathogenesis indicated a severe reduction in the inflammatory gene cascades. To test this further, *Ogg1*^−*/*−^ and control mice were subjected to an acute intestinal inflammatory challenge using dextran-sulfate-sodium-supplemented drinking water. In contrast to the lung pathology, the *Ogg1*^−*/*−^ mice showed a much more severe response than the wild-type controls, with greater weight loss, lethargy, diarrhea, and histology, consistent with severe ulcerative colitis. Consistent with the loss of functional *Ogg1*, GCMS analyses revealed significantly elevated levels of FapyGua, 8-oxoGua, and FapyAde [120]. Given the tissue-specific differences in inflammatory responses between the lungs and colon in *Ogg1*^−*/*−^ mice, additional investigations are warranted to harmonize these findings.

Since data from both the *Ogg1*^−*/*−^ and *Neil1*^−*/*−^ mouse models indicated that BER-mediated repair in mitochondria likely plays an integral role in regulating energy balance, we hypothesized that in contrast to decreased repair levels, increased expression of a mitochondrial-targeted human *OGG1* might render mice more resistant to obesity and metabolic syndrome [121–123]. These studies utilized a transgenic *OGG1* model (*OGG1*^*Tg*^) developed in the lab of Dr. Lars Eide, in which *OGG1*^*Tg*^ mice constitutively expressed the human *OGG1a* gene coupled with the mitochondrial targeting sequence of the manganese superoxide dismutase gene. These mice display modest, 2–3-fold increases in mitochondrial OGG1 activity above that observed in wild-type mice. This level of increase in the expression of the mitochondrial-targeted OGG1 is likely significant, because greatly elevated expression of OGG1 (or NTH1) increases cytotoxicity in lymphoblastoid cells [124,125]. These mice were challenged with the previously described high-fat diet, but extended for a total of 12 weeks. The *OGG1*^*Tg*^ mice were highly refractory to the development of obesity, insulin resistance, fatty liver, and adipose tissue inflammation, even though there was a significant increase in food intake in the *OGG1*^*Tg*^ mice. This reduction in body weight relative to wild-type mice was well correlated with increased energy expenditure in the *OGG1*^*Tg*^ mice. Additionally, examination of the histology of adipocytes in epididymal fat deposits in the *OGG1*^*Tg*^ mice versus controls revealed significant reductions in size, with concomitant large increases in the expression of genes regulating fatty acid oxidation and lipolytic processes. These changes in gene expression were restricted to white adipose tissue and strongly suggest that this organ is primarily responsible for the phenotypic changes associated with the *OGG1*^*Tg*^ mice. Thus, it is likely that the beneficial effects were achieved via a modest increase in the expression of mitochondrial-targeted OGG1, while a global large overexpression is detrimental [124,125].

While all of the above studies utilized a high-fat diet to accelerate the manifestations of metabolic syndrome, yellow Agouti mice (A^y^/*α*) represent a genetic model of obesity driven by hyperphagia. To test whether global expression of the human *OGG1* transgene could also confer resistance to weight gain in these mice, the A^y^/α were crossed with *OGG1*^*Tg*^ mice and selected A^y^/α^*Tg*^ [122]. Even though there were trends for increased food intake in the A^y^/α^*Tg*^ versus the A^y^/α, both weight and fat mass were significantly decreased in A^y^/α^*Tg*^ males and females. Consistent with the previous findings in the C57Bl6 background, the A^y^/α^*Tg*^ mice exhibited increased energy expenditure and mitochondrial content, without increased voluntary physical activity. Interestingly, the resistance to obesity and the increase in mitochondrial content in white adipose tissue were shown to be dependent on maternal transmission of the transgene, such that paternal transmission conferred no improvement in these parameters. These findings clearly suggest a major role for mitochondrial quality in determining resistance to obesity in offspring.

### 5.3. Inflammation: The Role of OGG1 as a Regulator of Transcriptional Responses to Oxidatively Induced Base Damage

In addition to its integral functions in the initiation of BER, an unanticipated role for OGG1 has emerged from in-depth analyses of phenotypes in the *Ogg1*^−*/*−^ mice relative to responses to inflammation-driven oxidative stress. A series of investigations using *Ogg1*^−*/*−^ mice revealed greatly reduced, selective airway gene expression levels in response to allergens [126,127] and resistance to lipopolysaccharide challenge, as well as resistance to tumor necrosis factor alpha (TNF-α)-induced inflammation [128,129]. Analyzed collectively, these data suggest that in the absence of Ogg1, this deficiency confers resistance to normal pro-inflammatory gene activation cascades. Key studies designed to understand the molecular basis of these observations have shown that under conditions of oxidative stress, 8-oxoG lesions preferentially form in promoter and gene-regulatory regions. Comparison of the sites of OGG1 and NF-κB binding following TNF-α mediated challenge revealed a major overlap [130]. This relationship is supported by data demonstrating that knockdown of *OGG1* significantly downregulates NF-κB-dependent gene expression profiles [127,131], suggesting that binding of OGG1 to sites of 8-oxoG does not initiate the chemistry of base release. This finding is consistent with data demonstrating that increased levels of 8-oxoG in GC-rich Sp1 DNA-binding sites are well correlated with the degree of gene expression enhancement in adipose tissue [132]. The mechanisms through which OGG1 locates and binds to damaged sites within genomic DNA have been extensively reviewed [31]. Furthermore, in the overall control of estrogen-responsive genes, chromatin binding and activation of the LSD1 demethylase produced localized oxidatively induced DNA damage which, in turn, recruited OGG1 and topoisomerase IIβ to these sites, thereby regulating estrogen-induced transcription [133].

The majority of these data are highly consistent with a model in which a catalytically incompetent form of OGG1 binds to an 8-oxoG site, and the lack of incision is achieved by either the modulation of the local DNA structure, rapid redox-driven post-translational modification, or association with other proteins. As discussed above, the potential roles of cysteine residues in OGG1’s function relative to its redox status have been recently addressed [76]. This non-catalytically active structure induces a sharp bend in DNA, facilitating the recruitment of NF-κB and its associated transcription initiation complex. Similar conclusions are likely germane for Sp1-regulated binding sites. Such non-catalytically productive binding of OGG1 to promoter and regulatory regions serves as the initiating step in activation by these master regulators. The re-establishment of pre-induction gene regulation in these regions could be achieved through a return to redox homeostasis and activation of OGG1-initiated BER.

In addition to the aforementioned examples of regulation in gene expression, several investigations have shown that the formation and processing of 8-oxoG lesions are key components in regions of the genome containing potential G-quadruplex-forming, Z-DNA-forming, and i-motif-forming sequences [53,134–137]. Interestingly, control of the expression of many DNA-repair genes is regulated in a strand-specific manner through structural alterations of potential G-quadruplex-DNA-forming sequences. Transitions in gene expression reflect transitions between the native, undamaged DNA and those containing 8-oxoG lesions that are processed by OGG1 and, subsequently, APE1.

Over the past 7 years, there have been significant advances in the development of small-molecule inhibitors of OGG1, and their use has substantiated conclusions drawn from genetic knockout models and potentially identified novel strategies for targeting OGG1 in disease prevention and treatment [138–141]. The lead compound developed within the Helleday laboratory—TH5487—which prevents binding of OGG1 to sites of base damage, has been successfully used to efficiently suppress allergen-induced inflammation and pro-inflammatory gene expression [140], attenuate pulmonary fibrosis [142], suppress polycystic ovarian syndrome by inhibiting NF-κB signaling [143], potentiate the anticancer efficacy of methotrexate [144], trigger synthetic lethality of olaparib in BRCA1 deficiencies [145], and limit inflammatory diseases [146]. Thus, it is evident that there are broad applications of OGG1 inhibitors in disease treatment, and more in-depth analyses are warranted to understand which function of OGG1 is responsible for these encouraging biological findings.

### 5.4. Roles of OGG1 and NEIL1 in Huntington’s Disease

Huntington’s disease is an autosomal-dominant, late-onset neurological disorder that is caused by expansion of CAG trinucleotide repeats in exon 1 of the *huntingtin* gene, resulting in increases in the length of a polyglutamine track from ~35 repeats in the wild type to >42 in fully penetrant disease (reviewed in [147,148]. Although oxidatively induced damage has been associated with various neurological diseases, as well as the aging process, work from Cynthia McMurray’s group reported the initial correlation between active repair of 8-oxo-dG lesions by OGG1 and an age-dependent expansion of CAG repeats—a process that they described as a “toxic oxidation” model [149]. In the development of this model, it was first established that there was an age-dependent, selective accumulation of oxidatively induced DNA damage in the brain and liver, even though repair levels did not appreciably decrease. Using hydrogen peroxide as an oxidative-damage-inducing treatment, it was demonstrated that there was a dose-dependent increase in single-stranded breaks, resulting in concomitant and selective expansion of the CAG repeats, while no expansion was detected in the TTC/GAA repeats. The essential role of OGG1 in initiating this process was established by demonstrating that CAG expansion was greatly reduced or delayed in most *Ogg1*^−*/*−^ mice relative to wild-type controls or *Nth1*^−*/*−^ or *Agg*^−*/*−^ mice. The key role of OGG1 in catalyzing the initiation of the trinucleotide repeat was confirmed using in vitro BER reconstitution assays, in which expansion occurred via repair-initiated strand displacement and slippage. Building on these investigations, Goula et al. [150] mechanistically expanded the role of BER in promoting triplet expansion by showing that both flap endonuclease 1 (FEN1) and a cofactor (HMGB1) were reduced in the striatum—a disease-susceptible brain tissue—while they were not reduced in disease-spared cerebellar regions. These data further established a mechanistic link between OGG1-initiated repair and CAG expansion in affected brain tissues.

As mentioned above, since triplet expansion was not completely abolished in *Ogg1*^−*/*−^ mice relative to wild-type controls, additional backup pathways have been examined for promoting the repair of oxidatively induced base damage. Specifically, Kovtun et al. [151] examined a potential role for the Cockayne syndrome B protein in transcription-coupled repair of such damage, along with knockouts of the mismatch repair proteins Msh2 and Msh3. Inclusion of analyses of Msh2 and Msh3 was well justified based on the role of mismatch repair also being well correlated with triplet expansion, with deficiencies in Msh2 and Msh3 leading to decreases in triplet repeats (reviewed in [152]). *Csb*^−*/*−^ mice showed a large reduction in deletions at CAG repeat tracts, while manifesting a fivefold increase in expansions. *Msh2*^−*/*−^ mice showed the mirror image of the *Csb*^−*/*−^ mice. Evidence was presented that CSB protects against CAG expansion by antagonizing the activity of Ogg1 and tract-length reduction during parent–child transmission.

At present, there are no therapeutically responsive treatments for Huntington’s patients, and there are ongoing questions as to whether inheritance of prior triplet expansions or somatic expansion is the primary driver of disease manifestation. To address this, murine models were created in which the CAG repeat in the murine *huntingtin* locus was expanded to contain a 150-repeat tract in the backgrounds of Ogg1 proficiency and deficiency [153]. These findings demonstrated that while the size of the inherited alleles did not influence somatic expansion, the loss of Ogg1 suppressed somatic expansion and the age of disease onset. Moreover, pharmacological intervention via the addition of a mitochondrial-targeted free-radical-scavenging agent suppressed somatic triplet expansion, with delays in the onset and progression of disease. These data provide the foundation from which novel therapeutic strategies can be developed.

Germane to Neil1’s role in maintaining genomic stability, expansion of trinucleotide repeats that are associated with the development of Huntington’s disease was significantly reduced in *Neil1*^−*/*−^ mice relative to controls [154]. Furthermore, this study demonstrated that Neil1 loss reduced germline expansion in males, concluding that Neil1 plays a role in germline and Huntington’s disease trinucleotide repeat expansion.

### 5.5. Neurodegeneration in Neil1 Deficiency

Due to the terminal replication status of neuronal cells, the potential adverse impacts of DNA damage are significant, with repair being the only viable option. In the aging brain, increased levels of oxidative stress and decreased repair can lead to cognitive declines and the manifestation of diseases such as Alzheimer’s disease (reviewed in [155]). Thus, it was anticipated that deficiencies in the repair of oxidatively induced damage in murine brains could be associated with various manifestations of brain dysfunction. For example, the *Neil1*^−*/*−^ mice showed impaired spatial memory retention and both increased brain damage and poorer outcomes in a focal ischemia/reperfusion stroke model [156]. *Neil1*^−*/*−^ mice also have overall deficits in olfactory function, as measured by decreased performances in multiple behavioral tests [157]. Further manifestations of neuronal deficits in these mice include the presentation of hyperactive, anxiety-mediated behaviors in open-field tests and deficiencies in cognitive memory as measured by novel object recognition [158,159]. To extend the scope of effects arising from Neil1 deficiency, sublethal ionizing radiation was administered to *Neil1*^−*/*−^ and control mice and tissues collected at different times [158]. Although both sets of mice lost weight post-radiation relative to non-irradiated controls, the decreases in the *Neil1*^−*/*−^ mice were more severe. Analyses of gene expression profiles in the brain cortex revealed that neuronal functional pathways were downregulated in radiation-treated versus sham-treated *Neil1*^−*/*−^ mice, while the opposite trend was evident in the wild-type mice. These irradiated *Neil1*^−*/*−^ mice showed decreased neurogenesis as measured by bromodeoxyuridine labeling. Non-irradiated *Neil1*^−*/*−^ mice displayed increased densities of microglia and astrocytes, suggestive of chronic neuroinflammation, as well as abnormal neuroinflammatory responses post-irradiation. Collectively, these data reveal an important role of Neil1 in brain development as well as the responsiveness of the brain to oxidatively induced stress, as evidenced by decreased neurogenesis, recovery from neuroinflammation, and behavioral deficits.

## 6. Extrapolation to Human Investigations

### 6.1. OGG1-Associated Cancer Variants

In contrast to the lack of carcinogenesis in murine models, epidemiological studies in human cancers show a positive correlation with the dominant human polymorphic variant of OGG1: Ser326Cys. In Asian and Caucasian populations, this variant is widely distributed, being present at 40–60% and 25–40%, respectively [160]. Biochemical characterization of the Ser326Cys OGG1 variant displays reduced catalytic efficiency, which may be partially attributable to its ability to form dimeric Cys-Cys disulfide bonds [161–163]. A significant number of adverse health effects have been reported in individuals carrying monoallelic Ser/Cys and biallelic Cys/Cys variants. These analyses have shown positive correlations with a variety of tumors, including colorectal cancer [164], breast cancer [165], oropharyngeal squamous-cell carcinoma [166], acute myeloid leukemia [167], endometrial cancer [168], prostate cancer [169,170], and lung cancer [171].

In addition to the S326C variant, R131Q—a catalytically inactive variant—has been associated with small-cell lung carcinoma [172]. Furthermore, R229Q has been reported to be associated with leukemia, with this variant demonstrating thermal lability and rendering cells sensitive to radiation [173,174].

### 6.2. Low-Level Expression of OGG1 in Therapeutically Responsive Subtypes of Human Acute Myeloid Leukemia (AML)

Within the diverse subtypes of human AML, there are two subtypes—RUNX1-RUNX1T1 fusion and CBFB-MYH11—that are significantly more therapeutically responsive than the remaining subtypes [175]. This increased efficacy is manifested in significantly longer disease-free survival. Analyses of the gene expression profiles of 404 genes (318 DNA-damage-response genes and another 87 additional genes related to DNA replication, the cell cycle, apoptosis, and Ara-C import and metabolism) revealed a unique gene (OGG1) that was downregulated in the cytarabine-responsive AML, but normal or elevated in the poor-response subtypes [176]. This investigation demonstrated that OGG1-deficient AML cell lines showed enhanced sensitivity to cytarabine treatment, with increased strand breaks occurring primarily at common fragile sites, along with enhanced oxidatively induced DNA damage. Insights into the mechanism of this enhanced lethality were guided by results that demonstrated that this preferential lethality could not be conferred by other replication-stress-inducing agents. However, increased cell killing was well correlated with a mechanism in which DNA polymerase δ inserted cytarabine opposite 8-oxo-dG, resulting in termination of DNA synthesis. The overall conclusion of this investigation suggested that the selective toxicity and therapeutic efficacy of cytarabine treatment in OGG1-deficient AML occurred via its incorporation opposite unrepaired 8-oxo-dG. These studies also suggest that selective small-molecule inhibitors of OGG1 could be used in combination chemotherapies for AML patients who are poorly responsive to standard-of-care chemotherapy.

### 6.3. NEIL1-Associated Cancers

It is currently unknown whether specific single-nucleotide polymorphic variants of NEIL1 are well correlated with increased carcinogenesis. Extrapolation of our investigations with *Neil1*^−*/*−^ mice, which displayed increased susceptibility to aflatoxin-induced carcinogenesis and increased DNA adduct levels, suggests that deficiencies in human NEIL1 might render individuals more susceptible to aflatoxin-induced HCCs. In this regard, a significant number of single-nucleotide variants of NEIL1 have been characterized for alterations in biochemical activities [44,47,71,177,178]. Directly relevant to HCC formation in portions of the world where aflatoxin exposure is very high, the three most common NEIL1 variants in East Asia—A51V, P68H, and G245R—have been biochemically characterized [178]. Although the catalytic efficiency of G245R was comparable to that of the wild-type enzyme, the activity of P68H was compromised—especially on the AFB_1_-FapyGua adduct. The A51V variant showed the ability to excise DNA adducts, but it was inactivated at 37 °C, with a half-life of ~5 min. Thus, A51V is predicted to be catalytically dead in a cellular context. The potential impact of these NEIL1 variants on susceptibility to early-onset HCC formation was addressed by genome sequencing of 49 aflatoxin-associated HCC patients in whom each of the three *NEIL1* variant alleles (i.e., A51V, P68H, and G245R) were identified, even though they were only present at 0.17%, 1.4%, and 0.65%, respectively, in this geographical region. To test the significance of these observations, expanded population-based studies are needed to ascertain whether there is an association between rare genetic variations in the *NEIL1* gene and susceptibility to HCC. Comparable analyses also need to be performed in other regions of the world that have endemic aflatoxin exposure and variants of NEIL1 that compromise catalytic efficiency and stability. Such investigations are warranted in sub-Saharan Africa, Turkey, and Central America (including Mexico).

### 6.4. Potential Applications of Agonists of OGG1 in Disease Prevention

In addition to the numerous correlations between the Ser326Cys OGG1 variant and cancer susceptibility, there are epidemiological studies implicating decreased OGG1 function in obesity and type 2 diabetes [179,180], Parkinson’s and Alzheimer’s diseases [181,182], and cardiovascular disease [183]. Collectively, these data suggest that there is therapeutic potential for the development of agonists of OGG1 in the treatment of human diseases. This suggestion is reinforced by the studies described above, demonstrating the health benefits in murine models with enhanced mtOGG1 expression. In this regard, the initial report of an agonist of OGG1 identified 8-bromoGua, which showed significant stimulation of the OGG1 lyase activity [74,184]. Additional studies have reported the identification of novel OGG1 agonists [163], with recent follow-up studies determining the underlying mechanisms of the AP lyase catalytic function and the benefits of enhanced mitochondrial repair [185]. Recently, another potential activator of OGG1 has been described in which the AP lyase activity of OGG1 was stimulated via small-molecule screening [186]. Overall, the development of agonists for OGG1 (and potentially other DNA glycosylases) is in very early development, with the identification of potential applications of these agonists still in an exploratory phase. In the coming years, the potential druggability of OGG1 for enhanced catalytic efficiency will likely be developed, and with those applications there should be a greater appreciation of the potential benefits of enhanced BER of oxidatively induced DNA damage.

## 7. Perspectives

Having the privilege of working in the field of BER—with particular emphasis on the DNA glycosylases—for over 40 years, it has been very gratifying to witness the progressive understanding of the function and diverse biological roles that these enzymes play in organismal health. This evolving perspective includes the target search mechanisms and chemistry through which base removal occurs, the ability to correlate catalytic efficiencies with glycosylase structure–function studies, the discovery of unanticipated phenotypes in murine knockout and transgenic models, and the potential applications of linking structure–function studies of single-nucleotide polymorphic variants with human disease. Despite all of this progress, there are still major challenges and unanswered questions within these areas of investigation. One such issue that deserves ongoing investigation concerns inter-laboratory discrepancies in murine phenotypes, including differences in spontaneous carcinogenesis in *Neil1*-deficient mice. A potential clue that may help resolve this challenge is the status and influence of the intestinal microbiome on organismal health, and how this differs between laboratories. Another area that deserves further investigation is organ-specific disease manifestations with respect to inflammation. Specifically, in the *Ogg1*^−*/*−^ model, the initiation of inflammatory cascades in these mice in response to oxidative stressors is significantly suppressed in the airway epithelial tissues and stomach, but enhanced in intestinal tissues. Developing a deeper understanding underlying these observations should result in a greater appreciation for the role(s) that OGG1 plays in the regulation of gene expression. In addition to the challenges, there are multiple areas representing opportunities in this field. This is especially germane with regard to clinical applications for personalized medicine related to disease susceptibility and the ability to create pharmacologically active small-molecule inhibitors and agonists of DNA glycosylases. Analyses of large-scale population- and disease-based sequencing, combined with systematic characterization of single-nucleotide variants, provide the opportunity to propose personalized medicine strategies to delay or prevent disease. This could entail dietary and lifestyle modifications, as well as vaccination when applicable. Finally, over the past 7 years, multiple laboratories and pharmaceutical companies have initiated screenings of different-sized libraries for inhibitors and, more recently, agonists of DNA glycosylases. As described herein, inhibitors of OGG1 may have broad applications in the treatment of human diseases, including asthma and acute myeloid leukemia. The potential health benefits associated with OGG1 agonists include suppression of weight gain and associated pathologies, as well as neurological diseases such as Parkinson’s and Alzheimer’s diseases. Overall, the robust history of past discoveries emanating from glycosylase-centric investigations bodes well for both fundamental basic scientific discoveries and potential applications with profound therapeutic benefits.
